# *In Vitro* Evaluation of the Biological Responses of Canine Macrophages Challenged with PLGA Nanoparticles Containing Monophosphoryl Lipid A

**DOI:** 10.1371/journal.pone.0165477

**Published:** 2016-11-11

**Authors:** Delphine Guldner, Julianne K. Hwang, Maria Clara D. Cardieri, Meaghan Eren, Parissa Ziaei, M. Grant Norton, Cleverson D. Souza

**Affiliations:** 1 Department of Veterinary Clinical Sciences, College of Veterinary Medicine, Washington State University, Pullman, Washington, United States of America; 2 School of Mechanical and Materials Engineering, Washington State University, Pullman, Washington, United States of America; Universidad de Castilla-La Mancha, SPAIN

## Abstract

Poly (lactic-co-glycolic acid) nanoparticles (PLGA NPs) have been considerably studied as a promising biodegradable delivery system to induce effective immune responses and to improve stability, safety, and cost effectiveness of vaccines. The study aimed at evaluating early inflammatory effects and cellular safety of PLGA NPs, co-encapsulating ovalbumin (PLGA/OVA NPs), as a model antigen and the adjuvant monophosphoryl lipid A (PLGA/MPLA NPs) as an adjuvant, on primary canine macrophages. The PLGA NPs constructs were prepared following the emulsion-solvent evaporation technique and further physic-chemically characterized. Peripheral blood mononuclear cells were isolated from canine whole blood by magnetic sorting and further cultured to generate macrophages. The uptake of PLGA NP constructs by macrophages was demonstrated by flow cytometry, transmission electron microscopy and confocal microscopy. Macrophage viability and morphology were evaluated by trypan blue exclusion and light microscopy. Macrophages were immunophenotyped for the expression of MHC-I and MHC-II and gene expression of Interleukin-10 (IL-10), Interleukin-12 (IL-12p40), and tumor necrosis factor alpha (TNF-α) were measured. The results showed that incubation of PLGA NP constructs with macrophages revealed effective early uptake of the PLGA NPs without altering the viability of macrophages. PLGA/OVA/MPLA NPs strongly induced TNF-α and IL-12p40 expression by macrophages as well as increase relative expression of MHC-I but not MHC-II molecules. Taken together, these results indicated that PLGA NPs with addition of MPLA represent a good model, when used as antigen carrier, for further, in vivo, work aiming to evaluate their potential to induce strong, specific, immune responses in dogs.

## Introduction

Vaccination is considered one of the most successful programs to control infectious diseases in human and veterinary medicine [1]. Not limited to the prevention of infectious diseases, vaccination strategies have recently been used as therapy for processes such as allergies, autoimmune diseases, and cancer [2–4]. Current vaccines rely mostly on inactivated pathogens or bacterial toxins, although problems related to the lack of purity, and thus safety, remain a challenge to overcome [1–2]. Recent research focused on the development of vaccines based on purified protein subunits, recombinant proteins, synthetic peptides and nucleic acids [1, 2, 4]. However, the higher purity of these novel antigens makes them poorly immunogenic requiring administration of adjuvants to help generating effective and robust immune responses [5, 6]. Adjuvants consist of any molecule that are capable of being recognized or to activate antigen presenting cells (APCs) thus aiding in the generation of effective humoral and cellular immune responses [1, 5]. Unfortunately, the current adjuvants are not effective for all antigens, can cause local undesirable reactions and generally fail to induce cellular immune responses; specifically cytotoxic T lymphocyte (CTL) responses [5]. Therefore, new approaches focusing on the development of more efficient and safer immunostimulants with the goal of achieving high and long-lasting immune responses are critically needed.

The emergence of nanoparticles (NPs) and their applications to medicine offer new opportunities for improved vaccine strategies [7–10]. Varied NP constructs are being explored as carriers for proteins, peptides, nucleic acids, and low molecular weight compounds [8–10]. Nanoparticles offer many advantages over more traditional approaches; it has been demonstrated that NPs facilitate the uptake of antigens by APCs, and improve antigen processing, presentation, and T cell priming [7, 9, 11, 12]. Among these molecules, Poly-lactic-co-glycolic acid (PLGA) NPs have been extensively studied in human medicine due to their excellent biocompatibility, safety and manipulability [7, 13, 14]. In addition, the slow nature of biodegradability of these PLGA NPs has been shown to sustain antigen delivery allowing a robust and effective adaptive immune response [14]. It has been shown that PLGA NPs, containing the model antigen OVA, are capable of eliciting a greater CTL response in human and mice compared to OVA with addition of incomplete Freud adjuvant [14, 15]. These studies offer encouraging insights into the development PLGA-derived NPs for preventive or therapeutic vaccination schemes against infectious diseases and cancer in veterinary medicine.

Characterization of the effects of PLGA NPs on the immune system necessitates *in vitro* evaluation of their interaction with APCs, as these cells are the first to interact with exogenous antigens. However, limited data is available regarding the effects of PLGA NPs on canine macrophages. Moreover, although few studies have been conducted, significant advances in cell magnetic sorting (MACS), and purification have provided fast and reliable methods to generate macrophages from peripheral blood of dogs which is expected to pave the way for further research in this area [16].

To our knowledge, the safety and effects of PLGA NP containing MPLA on the early innate-immune responses of canine macrophages have not been fully investigated. The present study first aimed at preparing PLGA NPs and PLGA NPs containing the model antigen OVA with addition of the TLR4 specific agonist monophosphoryl lipid A (MPLA). MPLA is a derivative of lipid A from gram negative bacteria, considerably less toxic than LPS whilst maintaining immunostimulatory activity *in vivo* and *in vitro* [17]. When tested in animal models as a vaccine adjuvant, MPLA facilitated inducing strong Th1 immune responses, which is desirable for effective immune-elimination of intracellular pathogens and neoplastic cells [18]. Subsequent aims for this project were to standardize the generation of monocyte-derived macrophages from dogs and to evaluate selected biological responses of macrophages when exposed to PLGA NP containing MPLA. We hypothesized that incubation of canine macrophages with PLGA NP preparations will trigger prompt uptake while eliciting negligible cellular toxic effects, and will stimulate immune responses characterized by increased gene expression of TNF-α and IL-12p40, and higher expression of MHC-I and MHC-II, particularly when MPLA is added to the formulation.

## Materials and Methods

### Animals and Ethics statement

Twenty healthy dogs with age ranging from 1 to 13 years (mean 5.5, ± 3.06), 7 females and 13 males of variable breeds from Veterinary Students and House Officers at the Veterinary Teaching Hospital at Washington State University were selected as a source of PBMCs for all experiments. The dogs were in good health, vaccinated, and regularly dewormed. An approval consent form was provided and signed by the owners of the animals involved in this study. This study was approved by The Institutional Animal Care and Use Committee (IACUC), at Washington State University under protocol number 04548-001/2015.

### Preparation of PLGA NP formulations

PLGA NPs were generated using the double emulsion water-in-oil-in-water (W/O/W) technique as previously described [7, 13]. Briefly, 9 milligrams of PLGA 75:25 (PolySciTech^®^, IN) of a molecular weight of 25–35 kDa was dissolved in 450 μL of chloroform and 50 μl of PBS was then added to create a water-in-oil (O/W) emulsion. The resultant solution was emulsified by sonication twice for 30 seconds each on ice using a Branson Ultrasonics Sonifier^™^ Cup Horn sonicator at an amplitude of 20%. This first emulsion was then added dropwise into 1 mL of 1% poly vinyl alcohol (PVA) (Sigma Aldrich, MO) of a molecular weight of 31–50 kD, while the mixture was being continuously vortexed. This second W/O/W emulsion was also sonicated three times for 40 seconds on ice at an amplitude of 40% and then the emulsion was added dropwise into 5 mL PVA (1%) and stirred for 4 hours to evaporate chloroform. Afterward, the mixture was centrifuged at 4°C for 15 minutes at 17,000 g. The supernatant was discarded and the PLGA NPs were washed three times with 5 mL of distilled water by bath sonication for resuspension and centrifugation at 4°C for 15 minutes at 17,000 g. After final washing the PLGA NPs were resuspended in 1 mL of water and freeze-dried. For preparation of PLGA NPs containing MPLA from S. Minnesota R595-TLR-based adjuvant vaccine grade^™^ (InvivoGen, CA), 50 μL of 2 mg/mL MPLA in 1:4 methanol-chloroform mixture was added to the PLGA polymer solution. For preparation of PLGA NPs containing MPLA with LPS-free Ovalbumin (InvivoGen, CA), 50 μL of 20 mg/mL OVA in PBS buffer was added to the PLGA-MPLA mixture. Lipopolysaccharide concentration analysis performed by LAL (Limulus amoebocyte lysate) before addition of MPLA to PLGA NPs showed negligible concentrations of LPS (data not shown).

### Determination of PLGA NP morphology

The morphology of the PLGA NPs were determined by scanning electron microscopy (SEM). Briefly, dried PLGA/OVA NPs and PLGA/OVA/MPLA NPs were applied onto a pin stub covered with double-sided carbon tape and then coated with 3.5 nm of platinum/palladium using a Cressington Sputter Coater. Images were taken using an FEI Quanta 200F SEM (Franceschi Microscopy and Imaging center, WSU, WA) at 5 to 10 kV accelerating voltage. The PLGA NP preparations sizes were measured using ImageJ software (NIH, 1.48v).

### Determination of PLGA NP polydispersibility and zeta potential

Polydispersibility was performed by the instrument Zetasizer nano ZS with DTS software (Malvern instruments, Worcestershire, UK). The PLGA NP formulations were taken in lyophilized form in microcentrifuge tubes, suspended in phosphate buffer, pH 7.4 and introduced in the instrument to read the results. Zeta potential of different formulations was measured by the instrument zetasizer nano ZS using DTS software. The experimental formulations were taken in lyophilized form in 2 mL Eppendorf tubes and the samples were suspended in phosphate buffer, pH 7.4 and then introduced in the instrument following manufacturer’s guideline.

### Determination of OVA and MPLA encapsulation efficiency

A micro-bicinchoninic acid (micro-BCA) protein assay kit (Pierce Biotechnology, Rockford, IL) was used for the determination of OVA loading (% wt) in the PLGA NPs. Accordingly, 2.4 mg of lyophilized PLGA/OVA/MPLA NPs were dissolved in 0.4 mL of 0.1 m NaOH solution. Following overnight incubation at 4°C, the antigen concentration was determined using a micro-BCA protein assay kit according to the manufacturer’s instructions for 96-microwell plates (Corning Inc., Corning, NY). The absorbance of the samples was measured at 562 nm using a microplate reader (EL808IU-PC, BioTek Instruments, Inc., Winooski VT). Blank PLGA NPs were used as control. The OVA encapsulation efficiency (EE) was calculated by the ratio of the OVA mass in the PLGA NPs over the total mass of OVA in the recipe. A Limulus Amebocyte Lysate (LAL) kit (Kinetic-QCL 192 test kit, 50-650U, LONZA) was used for the determination of the MPLA LC (% wt) in the PLGA NPs. Accordingly, aqueous MPLA solutions (0.01–10 ng/mL) were initially assayed with LAL using a microplate reader (BioTek EL808IU-PC) for establishing a calibration curve. The calibration curve was found to be linear over the MPLA concentration range of 0.01–10 ng/mL with a correlation coefficient of R2 = 0.9994. The mass of MPLA in the PLGA NPs was determined by subtracting the measured mass of MPLA in the four supernatants (collected after each washing cycle of PLGA NPs and diluted 10,000 fold in LAL reagent water) from the initial mass of MPLA in the recipe. The EE of MPLA was calculated by the ratio of the measured MPLA mass in the PLGA NPs over the total mass of MPLA in the recipe.

### In vitro release studies

The *in vitro* release of OVA from the PLGA/OVA/MPLA NP was carried out by incubating 1 mg of PLGA/OVA/MPLA NP in 1 mL of PBS at 37°C in a thermomixer (Thermomixer Compact, Eppendorf) at 400 g. At determined times (i.e., 1, 2, 4 weeks) PLGA/OVA/MPLA NPs were collected by centrifugation at 4,000 g for 15 min. The amounts of OVA released from the PLGA/OVA/MPLA NPs in the supernatant were measured by micro-BCA protein assay kit according to the manufacturer’s recommendations (as described above). The absorbance of the samples was measured at 562 nm using a microplate reader (BioTek Instruments, VT). Blank PLGA NPs were used as control.

### Blood sampling and monocyte isolation

Two to three dogs were used per experiment. Sixty mL of whole blood were collected from the jugular vein and were transferred to 50 mL tubes containing acid citrate dextrose (dilution 1:7). PBMCs were isolated by standard gradient centrifugation with Accu-paque^™^ density 1.088 (Chemical and Scientific Corporation, NY) as previously described [16, 19]. When erythrocytes were present in the PBMC sediment, the cells were further suspended in 2 mL of cold deionized water for 30 seconds to induce hemolysis. An average of 1x10^8^ cells/mL of PBMCs was typically obtained by using an automated cell counter (Moxi Z^™^, OS 4.0, Orflo^®^, ID). These cells were then processed by magnetic cell sorting (MACS, Miltenyi Biotec, German). The technique consists of incubating the PBMCs with 25 μL per 1x10^7^ cells/mL of monoclonal mouse anti-human CD14 (CAM36A, Monoclonal Antibody Center, Pullman, WA) at a concentration of 5 μg/mL during 15 minutes at 4°C with regular tube inversions. The cells were then incubated with goat anti-mouse IgG MACS microbeads (Miltenyi Biotec, Germany) following the manufacturer’s recommendations. Briefly, the cells were suspended in 40 μL of MACS buffer per 1x10^7^ cells and then incubated with 10 μL of IgG MACS microbeads at 4°C with regular tube inversions. The cells were then washed twice with MACS buffer. The CD14^+^ cells (i.e., monocytes) were further selected by magnetic sorting (Miltenyi Biotec, Germany). A total of 2x10^6^ cells/mL was usually obtained for downstream experiments.

### *In vitro* generation of primary cultures of monocyte-derived macrophages

CD14^+^ cells were cultured by adherence in a 12-well plastic culture plate with DMEM, high glucose, GlutaMAX^™^ supplement with HEPES medium (Life Technologies, CA). The cell culture media was supplemented with 10% fetal calf serum, 100 U/mL penicillin, 100 μg/mL streptomycin, and with 50 ng/mL canine recombinant GM-CSF (R&D Systems, MN). The cells were incubated for 7 days at 37°C and 5% CO_2_. Half of the cell culture media was changed after 3 days under culture conditions. A board certified clinical pathologist evaluated the *in vitro* proliferation and overall morphology of macrophages after 7 days in culture.

### Intracellular uptake of PLGA NP preparations by monocyte-derived macrophages

Canine macrophages were incubated with 50 μg/mL PLGA alone or PLGA NP containing LPS free OVA-FITC (OVA, EndoFit^™^, Invivogen, CA) for 24 hours. The cells were lifted after 10 minute-incubation at 37°C with a pre-warmed solution of 10 mM EDTA containing 5% fetal bovine serum. After two washes, the cells were fixed with 2% paraformaldehyde containing 2% glutaraldehyde in 0.1 M phosphate overnight at 4°C. The cells were dehydrated with ethanol: acetone solution and embedded in resin. Sections were realized via a microtome (Leica Reichert Ultracut R microtome, Franceschi Microscopy and Imaging Center, WSU, WA). The sections were then placed on a nickel grid, followed by staining with 2% aqueous uranyl acetate. Analysis of the uptake of PLGA/OVA-FITC/MPLA NPs by macrophages was performed by TEM (FEI Tecnai G2 20 Twin, Franceschi Microscopy and Imaging enter, WSU, WA) at 200kV.

Next we evaluated cellular uptake of PLGA NP/OVA-FITC with or without MPLA by confocal microscopy (Zeiss 510 META Confocal Laser Scanning Microscope, Franceschi Microscopy and Imaging Center, WSU, WA). Briefly, macrophages were cultured on coverslips in a 6-well-culture plastic plate and then incubated for 2 hours with 50 μg/mL of PLGA NP, or PLGA/OVA-FITC NPs, and PLGA/OVA-FITC/MPLA NPs. After cell culture media removal, the cells were fixed with 2% paraformaldehyde for 10 minutes at 37°C. The coverslips were rinsed with PBS and stained with a drop of ProLong ^®^ Gold antifade reagent with DAPI to be evaluated by confocal microscopy. DAPI (4',6-diamidino-2-phenylindole) was excited with a diode laser (405 nm) and the emission was collected with a BP420-480 IR filter. FITC was excited with an argon laser at 488 nm and the emission was collected with BP505-570 IR filter. Multi-tracking was used to decrease inter-channel cross talk. Confocal scanning parameters were set up for bright field background.

Further evaluation of phagocytosis was performed by flow cytometric analysis. Briefly, after 7 days of culture, 1 x 10^6^ macrophages/mL were treated with either PBS, 50 μg/mL OVA-FITC or PLGA/OVA-FITC/MPLA NP for 24 hours. Four wells of a standard 12-well plastic culture plate were assigned to each of these treatment groups. The cells were harvested using a sterile cell scraper, and thoroughly washed using PBS at 37°C to remove non-phagocytized PLGA NPs. Samples were acquired on a BD FACSCalibur and analyzed using FCS Express 4 (DeNovo Software, CA). Results were presented as histograms, with the macrophages incubated with PBS as a control.

### Viability of canine macrophage exposed to PLGA NP preparations

Macrophage viability was evaluated by trypan blue exclusion assay and morphology by light microscopy. Canine macrophages were incubated with 50 μg/mL PLGA NPs or 50 μg/mL PLGA/OVA/MPLA NPs for 2 hours or 24 hours. The adherent cells were harvested after incubation at 37°C with pre-warmed solution of 10mM EDTA/5% PBS and mixed with trypan blue (1:10). The percentage of viable macrophages, was assessed by a board certified clinical pathologist in a hemocytometer chamber using light microscopy. Aliquots of 1x10^6^ macrophages were further cytocentrifuged at 4000 g for 5 minutes (Cytospin^™^, ThermoFisher, MA), and stained with a Wright's-based stain. Thereafter, macrophage morphology was evaluated by light microscopy for evidence of apoptosis or cell necrosis by a boarded certified clinical pathologist.

### Determination of TNF-α, IL-12p40 and IL-10 expression by qRT-PCR

Seven day old canine macrophages were incubated with 50 μg/mL of PLGA NPs, PLGA/MPLA NPs, PLGA/OVA/MPLA NPs, or LPS (1 μg/mL) for 24 hours or 36 hours. Total RNA was extracted from the treated cells using the Aurum™ Total RNA Mini Kit (BioRad, CA) following the manufacturer’s recommendations for cultured cells. The amount of total RNA was determined spectrophotometrically (NanoDrop™ 1000 Spectrophotometer, Thermo Scientific, MA). Reverse transcription of mRNA into cDNA was processed in accordance with the manufacturer’s recommendations (iScriptTM Reverse Transcription Supermix for RT-qPCR, BioRad, CA) to obtain a cDNA final volume of 20 μL from 48 ng to 100 ng of total RNA using a thermal cycler (Axygen^®^ MaxyGene™ II Thermal Cycler, Corning, NY). The cDNA product was amplified by PCR using validated primers designed by RT2 qPCR Primer Assay (Quiagen, Germany). The sequences for which the primers were designed were canine IL-10 (RefSeq: NM_001003077.1), canine IL-12p40 (RefSeq: NM_001003292.1), and canine TNF-α (RefSeq: NM_001003244.4). Expression levels of GAPDH (RefSeq: NM_001003142.1) gene was used to normalize the input of cDNA. Real time PCR was performed using Power SYBR Green PCR Master Mix (Applied Biosystems, CA), following the manufacturer’s recommendations. Briefly, each well contained a 20 μL reaction mixture that contains 10 μl of the SYBR Green qPCR Master Mix, 4 μL of water, 2 μL of each specific primer set, and 4 μL of 1/20 diluted cDNA template. The thermal profile settings consisted of a 10-minute denaturation at 95°C, 40 cycles of 15 seconds at 95°C and 1 minute at 60°C and a dissociation stage at the end of the run from 60°C to 95°C with 0.3°C increments, and was carried out in a StepOnePlus^™^ Real-Time PCR system (Applied Biosystems, CA). At least two replicates of each product to verify for accuracy and a non-template control to test for contamination of assay reagents were included in each experiment. The relative quantity of target, normalized to an endogenous control and relative to a reference sample, was calculated based on the threshold cycle (Ct) value by using equation of 2 –^ΔΔCT^ using StepOne^™^ software (version 2.3).

### Immunophenotypic analysis of canine macrophages incubated with PLGA NP preparations

For a 24 hour period prior to collection, 1 x 10^6^ macrophage/mL were incubated with PBS, 50 μg/mL of PLGA NPs, and 50 μg/mL PLGA/OVA/MPLA NP in individual standard wells of a 12-well plastic culture plate. Three wells were assigned to each of the aforementioned study groups. The macrophages treated with PBS were utilized as a control. The cells were collected using a sterile cell scraper, and gently washed using PBS at 37°C. For phenotypic maturation studies, the macrophages of each group were separately incubated with 15 μg/mL of COLIS69A (mAb isotype control with specificity for E.coli J5), MHC-I (DH-H58A) and MHC-II (TH14B), followed by incubation with a fluorescein-conjugated IgG/IgM antibody. All mAbs were obtained from the Washington State University Monoclonal Antibody Center, Pullman, WA. Samples were acquired on a Becton Dickinson FACSCalibur (BD Biosciences, Franklin Lakes, NJ) by gating on the live cell populations. All non-CD14^+^ populations were excluded. The data was further evaluated using FCS Express 4 (DeNovo Software) and presented as dot plots.

### Statistical analysis

All experiments were run in biological triplicates. A two-tailed paired t-test was used to compare two data and when needed ANOVA analysis was used to compare more than two data sets. Values of *P<0*.*05* were considered statistically significant.

## Results

### Characterization of PLGA NP formulations

**Fig 1:**
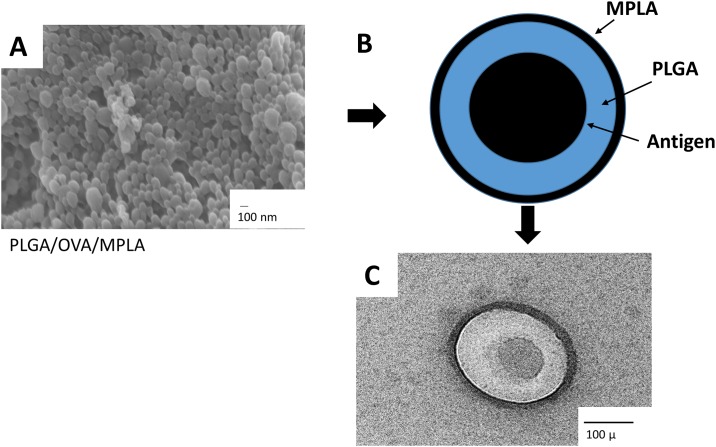
Characterization of PLGA NPs. (A) Morphological analysis of PLGA NP/OVA/MPLA by scanning electron microscopy (SEM). (B) Schematic representation of the PLGA/OVA/MPLA NP and (C) SEM of a single PLGA NP/OVA/MPLA. Scale bar = 100 nm and 100 μ. PLGA: poly-lactic-co-glycolic acid, MPLA: Monophosphoryl Lipid A, OVA: ovalbumin

**Fig 2:**
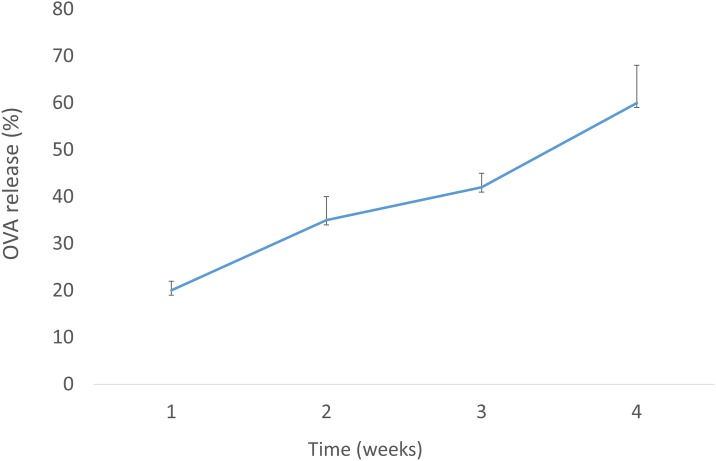
*In vitro* release profile of OVA from PLGA NPs. One milligram of the PLGA/OVA/MPLA NPs were dispersed in 4 ml of PBS containing 0.02% sodium azide, and then were kept at 37°C. At the indicated weeks, the supernatants were collected after centrifugation and the protein contents were analyzed.

Physical-chemical characterization, including hydrodynamic diameter, zeta potential, polydispersity index (PDI), mobility, conductivity, and encapsulating efficiency (EE) of OVA and MPLA, of the several PLGA NP constructs used in this study is summarized in Table 1. The mean diameters of PLGA, PLGA/OVA, PLGA/MPLA and PLGA/OVA/MPLA NPs were 431 nm, 658 nm, 400 nm, and 566 nm respectively. The zeta potential was negative for all NPs varying from −9.41 to −14.8 mV. The incorporation of the MPLA into PLGA NPs slightly reduced the overall negative surface charge of the NPs. The small differences in size and zeta potential between the different batches of PLGA NPS demonstrated the good reproducibility in the method of preparation used. The OVA EE for the PLGA/OVA NP and PLGA/OVA/MPLA NPs were 100% and 90.8%, respectively. High EE was also obtained for MPLA. Scanning electron microscopy of the PLGA/OVA/MPLA NP showed that the particles were spherical, presenting with smooth surface with absence of superficial irregularities containing an electro dense core when OVA was present (Fig 1). The in vitro release of PLGA/OVA/MPLA in PBS at 37°C was evaluated over 4 weeks (Fig 2). Results indicated that around 20% of OVA is released from PLGA/OVA/MPLA NPs during the first week reaching at maximum released profile at about 60% after 4 weeks. The obtained results are in agreement with those previously reported in the literature for the same PLGA NPs under similar conditions [7, 20].

**Table 1:** Properties of PLGA NPs constructs used in this study.

Formulation	Hydrodynamic diameter (nm)	Zeta Potential (mV)	Polydispersity Index	Mobility (umcm/Vs)	Conductivity (mS/cm)	OVA EE (%)^d^	MPLA EE (%)
**PLGA**^a^	431 ± 162	-14.8	0.141	-1.159	18	-	-
**PLGA/OVA**^b^	658.1 ± 273.7	-11.3	0.518	-0.885	19	100 ± 0.5	-
**PLGA/MPLA**^c^	400 ± 125	-8.58	0.947	-0.672	18	-	98.41 ± 0.09
**PLGA/OVA/MPLA**	566.7 ± 339.3	-9.41	0.358	-0.737	18	90.8 ± 0.1	97.34 ± 0.01

^a^ PLGA = poly (lactic-co-glycolic acid)

^b^ OVA = ovalbumin

^c^ MPLA = monophosphoryl Lipid A

^d^ EE = encapsulation efficiency

### Canine macrophage characterization

**Fig 3:**
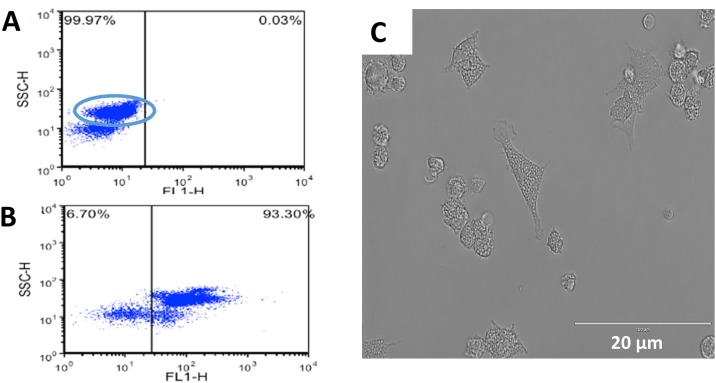
*In vitro* generation of canine monocyte-derived macrophages. Monocytes were obtained by positive selection using CD14 monoclonal antibody coated columns. CD14^+^ monocytes were then seed in 12 or 24 wells plates at 1x10^6^ cell/mL in DMEM enriched with 50 ng/mL of canine GM-CSF. Macrophages were allowed to mature for 7 days upon which were used for the experiments. Flow cytometry after monocyte isolation: (A) Control and (B) CD14^+^ cells. (C) Morphology of adherent macrophages after 7 days of incubation with DMEM and GM-CSF by light microscopy. Scale bar = 20 μm. Similar data were obtained in three independent experiments using macrophages from different animals.

Analysis by flow cytometry was performed to confirm that the cells collected after magnetic sorting were monocytes (CD14^+^). Briefly, the cells were incubated with a monoclonal mouse anti-human CD14 antibody. The cells were washed and further incubated with FITC goat anti-mouse IgG/IgM. The analysis by flow cytometry revealed that more than 93% of the mononuclear cells were CD14^+^ cells (Fig 3b). Monocytes were then incubated for 7 days at 37°C and 5% CO_2_ with addition of canine specific GM-CSF and appropriate culture media. Further evidence of *in vitro* generation of macrophages was assessed by light microscopy based on their large size, cytoplasmic granularity and cell appendages (Fig 3c). Light microscopy analysis, by a board certified clinical pathologist, of the macrophage culture showed no appreciable lymphocyte contamination (<1%) after 7 days in culture (data not shown).

### PLGA NP uptake by canine macrophages

**Fig 4:**
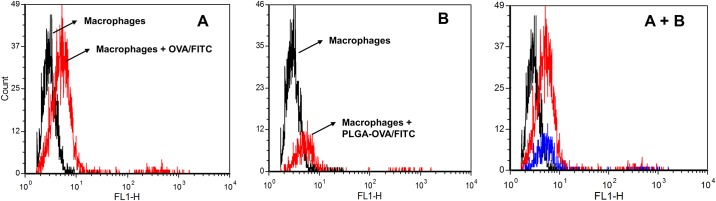
Flow cytometric evaluation of cellular uptake of PLGA NP preparations by canine macrophages. Flow cytometry analysis of canine macrophages treated with fluorescent PLGA/OVA/MPLA NPs. Day 7 canine macrophages cultures (1x10^6^) were incubated with 50 μg/mL of OVA-FITC or PLGA/OVA-FITC/MPLA NP. After 24 hours, nonadherent cells were harvested and analyzed by flow cytometry. One representative experiment is shown. (A) Histogram of macrophages incubated with 50 μg/mL OVA-FIT for 24 hours. (B) Histogram of macrophages incubated with 50 μg/mL PLGA/OVA-FITC/MPLA for 24 hours.

**Fig 5:**
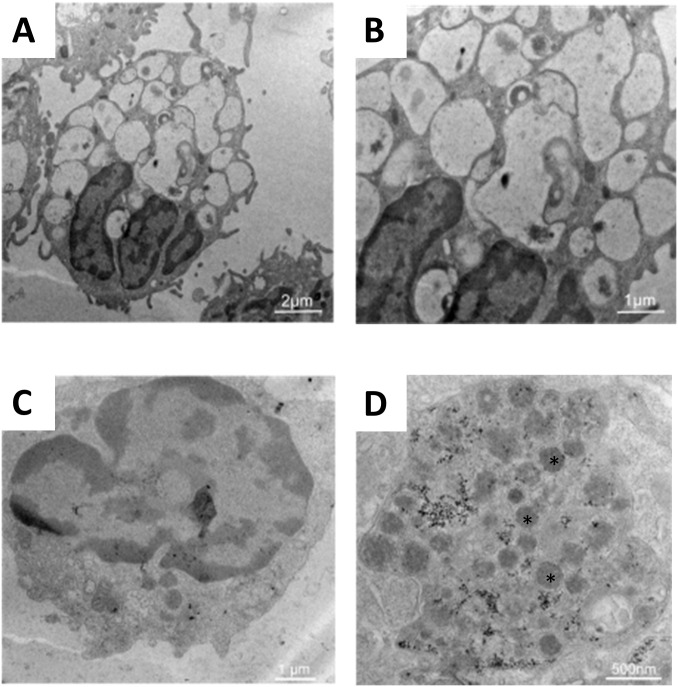
Transmission electron microscopy (TEM) evaluation of cellular uptake of PLGA NP preparations by canine macrophages. Day 7 canine macrophages cultures (1x10^6^) were incubated with 50 μg/mL of PLGA/OVA/MPLA NPs. After 24 hours, nonadherent cells were harvested and analyzed by TEM. The macrophages without treatment (A) and (B) contain empty phagocytic vacuoles while the macrophages incubated with PLGA NP/OVA/MPLA (C) and (D) demonstrated absence of vacuoles and the presence of electron-dense material reflecting uptake of the NPs. (A) scale bar = 2 μm, (B) and (C) scale bar = 1 μm, and (D) scale bar = 500 nm. Similar data were obtained in three independent experiments.

**Fig 6:**
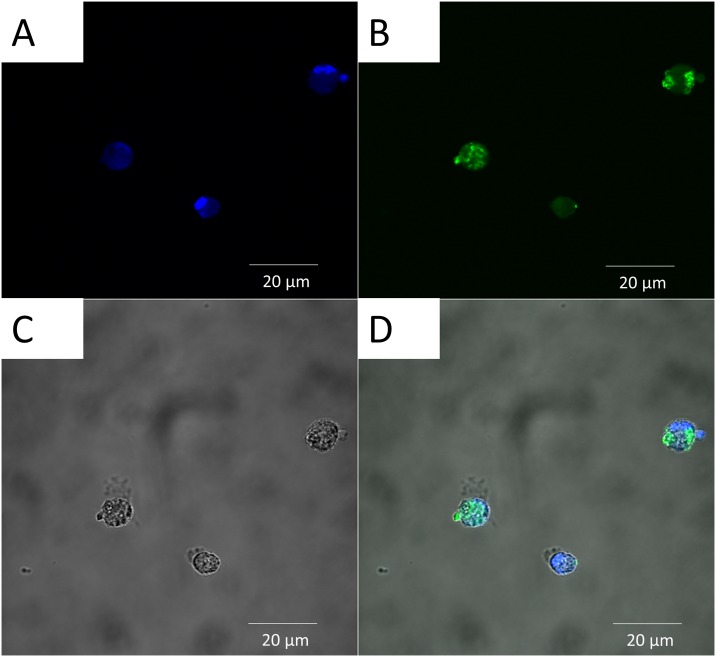
Cellular localization of PLGA/OVA-FITC/MPLA NP uptake by canine macrophages and evaluated by confocal laser scanning microscopy after 2 hours of incubation. Day 7 canine macrophages cultures (1x10^6^) were incubated with 50 μg/mL of PLGA/OVA-FITC/ MPLA NPs. After 2 hours, macrophages were harvested and analyzed by confocal microscopy. One representative experiment is shown. (A) Nucleus of macrophages stain blue by DAPI. (B) PLGA/OVA-FITC/MPLA NP stain green. (C) Canine macrophages. (D) Merge of A&B demonstrating internalization of particles. Scale bar = 20 μm. DAPI = 4',6-Diamidino-2-Phenylindole, Dihydrochloride; FITC = Fluorescein isothiocyanate. Similar data were obtained in three independent experiments.

Phagocytosis of antigens is one of the first steps necessary for an effective immune response. Therefore, assessment of this process is paramount when evaluating new vaccine protocols and newer antigen delivery systems. The cellular uptake of the PLGA NP preparations by canine macrophages was evaluated by flow cytometry, TEM and confocal microscopy. Seven day old canine macrophages were incubated with PLGA/OVA-FITC/MPLA for 24 hours and mean fluorescent intensity (MFI) of FITC was measured by flow cytometry against macrophages incubated with PBS or OVA-FITC (i.e., positive control). Results showed that PLGA NP were efficiently internalized by canine macrophages although less efficiently when compared to control (Fig 4). The lesser fluorescence observed in this study might be caused by earlier denaturation of OVA/FITC as part of the normal antigen presentation process or that the incubation interval need to be longer for optimal phagocytosis. Nevertheless these observations were consistent with previous studies performed on the ability of APCs to efficiently phagocytize PLGA-derived NPs [7, 21, 22]. Transmission electron microscopy studies demonstrated that macrophages incubated with PLGA/OVA/MPLA NP for 24 hours displayed several electron dense intra-cytoplasmic structures in comparison to the control (i.e., PBS alone), consistent with internalization of the PLGA NPs (Fig 5c and 5d). In addition to flow cytometry and TEM, the cellular localization of PLGA NPs was assessed by confocal microscopy (Fig 6). The nucleus of macrophages stained in blue due to the fluorescence emitted by DAPI (Fig 6a). The green fluorescence is emitted by the internalized PLGA/OVA-FITC/MPLA NP (Fig 6b). By merging the colors, the PLGA/OVA-FITC/MPLA NPs are shown to be localized within the cytoplasm of the macrophages (Fig 6d). Comparable results were obtained when PLGA OVA-FITC without MPLA NPs were used (data not shown), suggesting that MPLA had no effect on the ability of canine macrophages to uptake the several PLGA NP formulations used in this study. Overall, these results indicate that PLGA/OVA/MPLA NP are promptly and efficiently phagocytosed by canine macrophages and that future *in vivo* studies, to assess potential for induction of effective immune responses, is warranted.

### Macrophage viability

**Fig 7:**
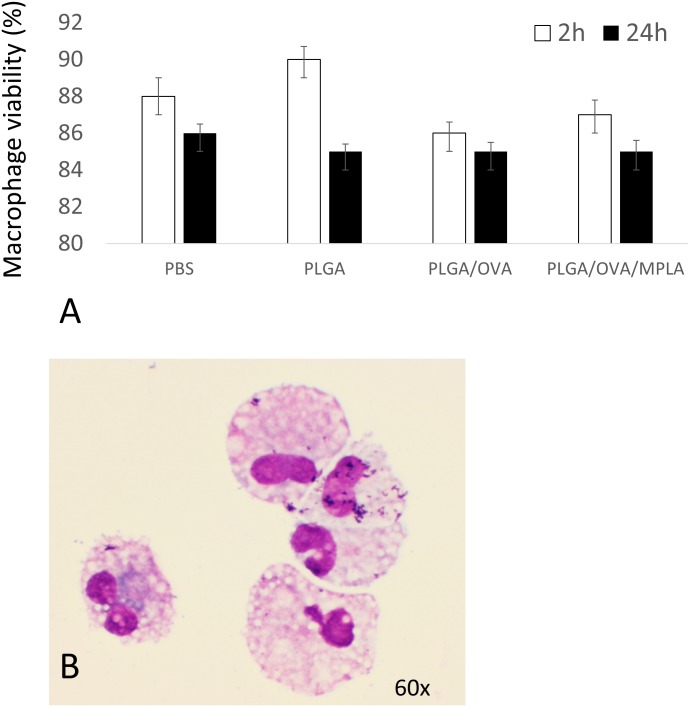
Evaluation of canine macrophage viability incubated with PLGA NP formulations. Day 7 canine macrophages cultures (1x10^6^) were incubated with 50 μg/mL of different NP formulations for 2 hours or 24 hours at 37°C. Macrophages were then stained with Trypan Blue and evaluated microscopically. (A) Cells that take the stain have impaired membranes and are considered dead cells. (B) Day 7 canine macrophages (1x10^6^), were cytocentrifuged and evaluated under light microscopy for evidence of pyknosis or cellular necrosis. Similar data were obtained in three independent experiments.

Macrophage viability was evaluated by incubating canine macrophages with PLGA/OVA NPs and PLGA/OVA/MPLA NPs for 2 hours or 24 hours. Briefly, the macrophages were lifted from the culture plates with warm PBS, washed three times, and mixed with the trypan blue stain in a proportion of 1:10. The percentage of viable macrophages in the control group (i.e., PBS) after 2 hours and 24 hours were 88.5% (± 0.05) and 85.5% (± 0.8), respectively and incubation with empty PLGA at the same time points were 89% (± 0.04) and 85% (± 0.03), respectively. The viability of macrophages incubated with PLGA/OVA NPs were 85.6% (± 0.1) and 84% (± 0.1) and with PLGA/OVA/MPLA NPs were 86.8% (± 0.8) and 84.3% (± 0.9), respectively. The results showed that there were no significant differences (*P-value <0*.*05*) between the control group, PLGA/OVA NPs, and PLGA/OVA/MPLA NPs, at 2 hours or 24 hours post incubation (Fig 7a). In addition, cultures of 7 day old macrophages incubated with different PLGA NP constructs for 24 hours and evaluated by light microscopy by a boarded certified clinical pathologist demonstrated lack of abnormal cytomorphological features consistent with cell dead (i.e., apoptosis and necrosis) as illustrated in (Fig 7b). Lack of toxicity of PLGA/OVA with or without MPLA NPs to APC was expected and corroborate with several studies performed previously [13, 15].

### Cytokine expression

**Fig 8:**
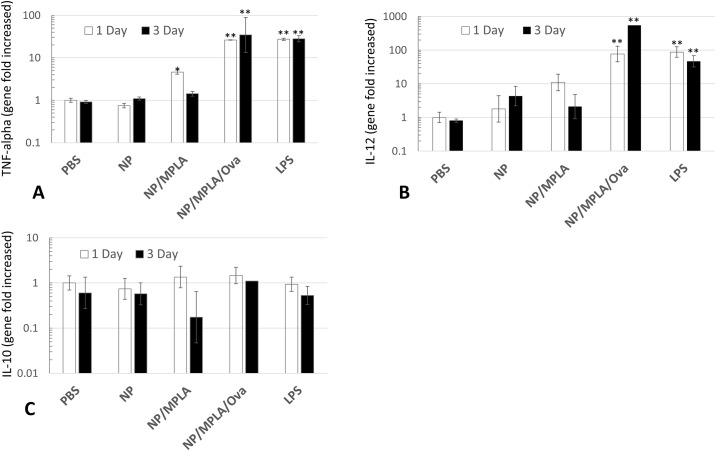
Effects of incubation of PLGA NP preparations on TNF-alpha (A), IL-12p40 (B), and IL-10 (C), gene expression by canine macrophages. Day 7 canine macrophages (1x10^6^/mL) were pretreated with 50 μg/mL of PLGA NP, PLGA NP/MPLA, PLGA NP/OVA/MPLA or LPS (1 μg/mL) for 24 hours or 36 hours. GAPDH was used to normalize the results. * Statistically significant difference when compared to PBS and PLGA NP-treated group (*P<0*.*05*). ** Statistically significant when compared with PLGA NP/OVA/ MPLA treated macrophages (*P < 0*.*05*). Similar data were obtained in three independent experiments.

Inflammatory and pro-immune cytokine release by activated professional phagocytes are important components of an effective immune response. In this study, pro-inflammatory (i.e., TNF-α), pro-immune (i.e., IL-12p40) and anti-inflammatory (i.e., IL-10) cytokine expression were measured to evaluate the potential of PLGA NP preparations to induce early, protective, immune responses initiated by macrophages. Canine macrophages were incubated with different PLGA NP constructs and the expression of the above cytokines were determined by qRT-PCR at 24 hours and 72 hours (Fig 6). The results showed that canine macrophages incubated with PLGA/MPLA NPs and PLGA/OVA/MPLA NPs express significant amounts of TNF-α at 24 hours and 72 hours when compared to PBS and PLGA NP alone (Fig 8a). Interestingly, only PLGA/OVA/MPLA NPs stimulated greater amounts of TNF-α which was comparable to the expression elicited by incubating macrophages with bacterial LPS. Similarly, PLGA NP preparations induced significant amounts of the cytokine IL-12p40 expression at 24 hours and 72 hours only when MPLA and OVA were present on the formulation (Fig 8b). Importantly, expression of IL-10 did not appear to be induced by incubation of canine macrophages with any of the PLGA NP formulations used in this study (Fig 8c). Taken together these results indicates that PLGA/OVA/MPLA NPs have the potential to induce robust protective inflammatory responses that might be translated to effective immune responses, although this assumption needs to be demonstrate *in vivo*.

### Flow cytometry analysis of MHC-I and MHC-II expression by canine macrophages incubated with PLGA NPs

**Fig 9:**
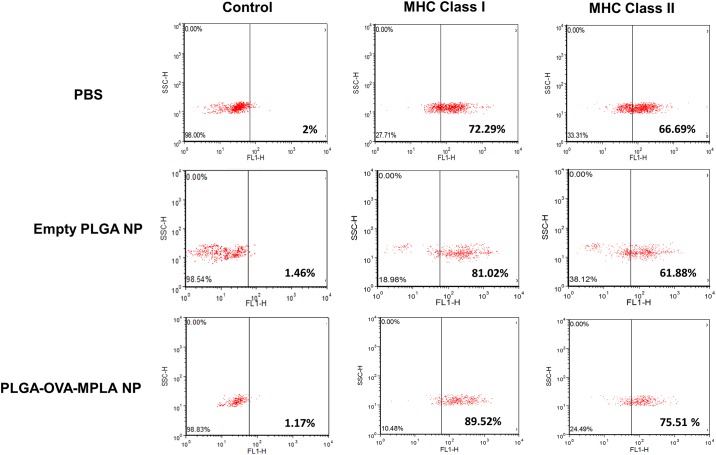
Flow cytometric evaluation of MHC-I and MHC-II phenotype in canine macrophages exposed to PLGA NP preparations. Day 7 canine macrophages (1x10^6^) were incubated with 50 μg/mL of PLGA NP or PLGA/OVA/MPLA NP. After 24 hours, nonadherent cells were harvested and analyzed by flow cytometry. FSC (forward scatter) and SSC (side scatter) profiles of PBS and PLGA NP-incubated with canine macrophages are shown. One color flow cytometry dot plots gated indicating the phenotype of macrophages incubated with PLGA NPs. One representative experiment is shown.

In order to determine the effects of PLGA NPs upon phenotypical properties of canine macrophages, flow cytometry was used to quantify the expression of molecules involved in antigen presentation (i.e., MHC-I and MHC-II). Seven day old macrophages were stimulated with PBS, empty PLGA NP or PLGA/OVA/MPLA NP for 24 hours and thereafter cells were evaluated by flow cytometry. Baseline control canine macrophages demonstrated 72.29% and 66.69% expression of MHC-I and MHC-II, respectively. Incubation with empty PLGA NP and PLGA/OVA/MPLA NP induce up-regulation of MHC-I when compared to control at 81.02% and 89.02%, respectively (Fig 9). Interestingly, MHC-II was only mildly up-regulated by incubating macrophages with PLGA/OVA/MPLA at the time point investigated in this study. These results corroborated with previous research and indicate that the model studied here could be further evaluated for its potential to stimulate T cell responses against tumors or intracellular pathogenic organisms in dogs [15, 23].

## Discussion

Designing effective vaccines and immunotherapeutics against infectious diseases and cancer remains a major challenge in human and veterinary medicine. Important components of successful immune-protectants include the nature of the delivery system, the antigen immunogenicity and the adjuvants used—all of which are crucial for generating potent, effective and long-lasting immune-protection. Professional phagocytes, like macrophages and DCs, plays an important role initiating early innate-immune responses and priming the immune system for development of effective adaptive-immune responses. In the present study, we evaluated the effects of PLGA/OVA NPs, containing the immune-stimulant MPLA, on canine monocyte-derived macrophages *in vitro*. The study was conducted to characterize a novel antigen delivery system candidate that could be used, in future studies, in designing newer immunotherapeutics and vaccines in companion animals.

PLGA NPs were prepared with the emulsion-solvent evaporation technique which is the most efficient method to prepare PLGA NPs [24, 25]. In order to encapsulate the model antigen OVA and MPLA, a modified technique, the double emulsion water-in-oil was performed as previously described [26]. The PLGA NP constructs produced had smooth, regular surfaces, were mostly non-aggregating, with optimal antigen encapsulation, and had a particle size that was conducive to uptake by macrophages. All these characteristics indicated that the PLGA NP formulations developed were suitable for use in the assays described in this study.

Purification of CD14^+^ monocytes from PBMCs were performed by magnetic sorting instead of the more traditional plastic attachment method to avoid potential lymphocyte contamination. Use of flow cytometry confirmed that the majority (i.e., >93%) of the cells isolated from canine PBMCs were CD14^+^ monocytes. Incubation of CD14^+^ monocytes with canine recombinant GM-CSF with high glucose DMEM culture media for 7 days, yield a population of macrophages with defined morphology and minimal lymphocyte contamination. These results corroborate with a previous study where canine monocyte-derived macrophages were successfully generated by magnetic sorting following incubation with canine recombinant GM-CSF, although the media conditions used were different [16].

Nanoparticles must have comparable dimensions to bacterial pathogens to be fully recognized by the immune system. Particle size has been shown to be important for APC internalization and intracellular processing [24, 27]. According to published research, particles of 20–200 nm are usually taken up via endocytosis by APCs, while those with 0.5–5μm are primarily taken up by macrophages via macro-pinocytosis or phagocytosis [15]. In addition, it has been demonstrated that the smaller the PLGA NPs used to deliver the antigen the stronger the antigen-specific CD8^+^ T cell response produced in mice [15]. To evaluate the cellular uptake of PLGA NPs by canine macrophages, we incorporated a model antigen OVA conjugated to FITC to our PLGA NP preparation. Transmission electron microscopy confirmed the presence of electron dense particles within the cytoplasm of the canine macrophages exposed to PLGA/OVA-FITC NPs at early time points post incubation. In addition, confocal laser scanning microscopy was used to detect the fluorescence emitted by PLGA/OVA-FITC NPs. Our results showed that PLGA/OVA-FITC NPs and PLGA/OVA-FITC/MPLA NPs were localized within the cytoplasm of the majority of macrophages 2 hours post treatment *in vitro*. Flow cytometry experiments also indicated that most macrophages phagocytosed PLGA/MPLA NPs within 24 hours post exposure. Taken together, these experiments demonstrated that canine macrophages were capable of efficiently uptaking PLGA/OVA-FITC NPs and PLGA/OVA-FITC/MPLA NPs at early time points post incubation. The presence of MPLA seems not to alter the phagocytic activity of macrophages. These findings suggest that canine macrophage activation would start rapidly after exposure with PLGA-derived NPs *in vivo* as has been demonstrated in mice and human experiments [7,15].

Macrophage viability, evaluated by the trypan blue assay, yielded a high percentage of viable macrophages after 2 hours and 24 hours of incubation with PLGA NP preparations. No statistical significant differences were observed when PLGA/OVA NPs and PLGA/OVA/MPLA NPs treated macrophages were compared to the control (i.e., PBS) at 2 hours and 24 hours. In addition, morphological evaluation of 7 days old macrophages incubated with different PLGA NP preparations for 24 hours did not demonstrate changes associated with cell death including apoptosis and necrosis. These results suggested that PLGA NP with or without addition of MPLA are safe to use in *in vivo* experiments and corroborates with other similar studies [7, 15, 21, 22].

Activation of APCs and subsequent priming of lymphocytes is essential for an effective immune response against exogenous or endogenous antigens. APCs activate adaptive immune responses primarily by presenting antigens thought MHC-I and MHC-II to CD8^+^ and CD4^+^ lymphocytes, respectively. Inflammatory and pro-immune cytokine production by APCs and lymphocyte subsets is also needed for a complete and robust response. Here, we evaluate early cytokine and MHC-I and MCH-II expression by canine macrophages stimulated with PGLA NP preparations. We chose to measure a pro-inflammatory cytokine, a pro-immune cytokine, and an anti-inflammatory cytokine to evaluate the potential of PLGA NP preparations to stimulate early, protective, immune responses. This evaluation is necessary as part of the general assessment of using PLGA NPs as a vaccine delivery system candidate in future *in vivo* studies in dogs.

Secreted soluble TNF-α has been previously detected at early time points after RAW264 macrophages stimulation with LPS [28]. This cytokine acts as pro-inflammatory molecule activating the innate and adaptive immune cells resulting in further production of pro-inflammatory mediators [28]. At physiological concentrations, TNF-α helps induce protective immune responses and inflammation which are beneficial to the host. Interleukin-12 has been described as an immunostimulatory cytokine, predominantly by driving the differentiation of naïve T cells into protective Th1 cells [29, 30]. It has also been shown to stimulate the production of IFN-γ and TNF-α from T cells subsets and natural killer (NK) cells, and to reduce IL-4 mediated suppression of IFN-γ [31]. Our results showed that both TNF-α and IL-12p40 were upregulated as soon as 24 hours when macrophages were stimulated with PLGA NPs containing MPLA. Interesting, addition of OVA to the PLGA NP preparation containing MPLA stimulated greater expression of TNF-α and IL-12p40 at 24 hours and 72 hours compared to the control and PLGA/MPLA NPs. The level of expression was comparable when macrophages were stimulated with bacterial LPS suggesting that this formulation (i.e., PLGA/OVA/MPLA NP) is capable of eliciting strong inflammatory and pro-immune responses [14, 15, 17, 28, 32]. We postulated that the model antigen OVA may stimulate intracellular innate receptors like NOD1/2 or cytosolic TLRs which could potentially cause an indirect effect on TNF-α and IL-12p40 expression. An alternative explanation would be that OVA potentiates the interaction or engagement of MPLA with its ligand TLR4. Further experiments where OVA LPS-free is incubated with canine macrophages could be pursuit in the future to further understand this phenomena.

Interleukin-10 is known to suppress macrophage activation and to down-regulate inflammatory and antimicrobial responses such as the production of nitric oxide (NO), TNF-α, and IL-12. This cytokine also antagonizes the effects of IFN-γ and suppresses the activation of Th1-type cells by APCs [33]. Thus, IL-10 has a key effect on the limitation of the immune responses induced by APCs by inhibiting the production of key molecules involved in the establishment of Th1 immune responses. Such specific immune responses are needed for immune-elimination of intracellular pathogens and cancer cells. Here, incubation of canine macrophages with PLGA NP preparations containing MPLA and OVA failed to stimulate expression of IL-10 at 24 hours and 72 hours. It would be interesting to evaluate the IL-10 expression using longer incubation times, as IL-10 has been shown to be expressed at later time points after LPS or mycobacterial stimulation [19, 25]. Importantly, absence of significant secretion of IL-10 by APCs upon stimulation with PLGA-derived vaccine carriers would be beneficial as immune-modulatory molecules like IL-12 could stimulate more effective Th1 immune responses.

The major histocompatibility complex (MHC) is a cluster of genes that are important in the immune response. Major histocompatibility complex gene products interact with bound peptides and present these antigens to T-cells via interactions with the T-cell receptors. This presentation of antigens to the immune system is crucial in the regulation of the immune responses [34]. Efficient antigen presentation to T cells encapsulated by PLGA NPs to APCs have been studied. One study with human DCs loaded with PLGA-NPs encapsulating tumor peptide induced significantly stronger cytotoxic T cell responses than those pulsed with free tumor peptide. An important finding of that study was that the tumor peptide dose encapsulated in PLGA-NPs was 63 times less than that emulsified in incomplete Freund's adjuvant, but it induced a more potent cytotoxic T cell response *in vivo* [15]. Here, canine macrophages incubated with PLGA/OVA/MPLA NP induced up-regulation of MHC-I when compared to control. Interestingly, MHC-II was consistently only mildly up-regulated by incubating macrophages with PLGA/OVA/MPLA at the time point investigate. These results indicated that the model studied here could be further evaluated for its potential to stimulate cytotoxic T cell responses against tumors or intracellular pathogenic organisms in dogs.

In summary, we were able to successfully generate and characterize different PLGA NP constructs and canine monocyte-derived macrophages *in vitro*. Our results showed that PLGA/OVA/MPLA NPs are promptly and efficiently phagocytized by canine macrophages. Addition of MPLA to the formulation of PLGA/OVA NP strongly stimulated expression of TNF-α and IL-12p40, with lesser amounts of IL-10 expression along with increased expression of MHC-I by canine macrophages. Taken together these results indicated that PLGA-derived NPs with addition of MPLA represent a good model for further *in vivo* characterization as a new antigen carrier capable of stimulating effective immune responses in dogs.
